# Book Review

**Published:** 1909-12

**Authors:** 


**A Manual of Medical Treatment, or Clinical Therapeutics.** New Edition. By I. BURNEY YEO, M.D., RAYMOND CRAWFURD, M.A., M.D., and E. FARQUHAR BUZZARD, M.A., M.D. Two Volumes. Pp. xv, 802; viii, 829. London: Cassell & Co. Limited. 1909.—Previous reviews of the original editions of these books have appeared in Vols. XII and XX. The original plan has been continued, but the work has undergone thorough revision. and the author has added the name of Dr. Crawfurd—who undertook to revise the sections on digestive and circulatory diseases, as well as those dealing with diseases of the liver and kidneys—and that of Dr. Buzzard, whose special knowledge and experience of diseases of the nervous system will add greatly to the value of parts dealing with these diseases. The work has been so much appreciated in the past, that this new and revised form of it neither needs nor deserves criticism or commendation.

**The "Nauheim" Treatment of Diseases of the Heart and Circulation.** By LESLIE THORNE THORNE, M.D. Third Edition. Pp. xi, 82. London: Baillière, Tindall & Cox. 1909.—The original edition is somewhat enlarged by the addition of a full description of the "Schott" Exercises, with twenty-eight photographs of the various phases. The author considers that this edition contains all the details required to enable those unacquainted with the treatment to administer both the "Nauheim" Baths and the "Schott" Exercises. We think, however, that the skilled experience of those trained to the work must be better than the experiments of the tyro, and it is frequently the case that the experimental learner fails to obtain the marvellous results which are commonly reported by the enthusiasts of the Nauheim methods.

**Health, Morals and Longevity.** By George Gresswell, M.A., L.R.C.P. & S. Ed., and Albert Gresswell, M.A., M.D., M.R.C.S. Pp. xii, 229. Bristol: John Wright & Sons, Ltd.—The purport of this book, as stated in the preface, is to give a "short *résumé* of the more important conditions of living a healthy life." This naturally leads the authors into many fields of inquiry. They touch upon morality, conduct, the germ theory, notification of infectious diseases, old age, pain, sleep, nutrition, cleanliness, death, and other important subjects. The views of many philosophers and scientific workers are quoted, and interesting statistics are given as to the duration of life in different animals and plants, and so forth. Such questions as immunity, phagocytosis, insomnia and narcolepsy are not forgotten, so that the reader has a wide choice, and can browse on many pastures. An immense amount of diligence and labour have been spent on the book, and it may serve a useful purpose as a work of reference of a somewhat superficial sort; for the list given of the subjects discussed is sufficient to show that not very much space can be given to each. It may be described as a compendium of current views on many frequently recurring and interesting questions.

**Operative Nursing and Technique.** By Chas. P. Childe, F.R.C.S. Pp. xiii, 224. London: Baillière, Tindall & Cox. 1909.—This little book of 217 pages is a model of clearness and conciseness. It relates, in an exact and detailed manner, the author's requirements of his nursing staff. The method of attaining asepsis is thorough and logical, though perhaps there is here some unnecessary repetition. The description of nursing is almost confined to that of abdominal cases, but this is exceedingly good. A chapter on instruments is hampered by the difficulty of selection of the important from the unusual and rare ones. The illustrations are clear and well selected, and the book is one which ought to be accessible to every nurse during her training.

Occasionally the imagination is unintentionally stimulated, as where it is suggested that for the examination of the throat "small wooden spatulas, or *even the child's own pocket handkerchief*, may be used to depress the tongue"; or again, where in describing a surprise visit to his school, he calls up the vision of the doctor placing his benevolent hand on the once verminous, but now clean and close-cut hair of a little girl, and so ably commending her to her schoolfellows that they rush home and clamour for a crop! Truly a delightful picture; would it were as delightfully true.

**A Manual of Operative Surgery.** By Sir Frederick Treves, Bart., and Jonathan Hutchinson, F.R.C.S. Third Edition. Vol. I. Pp. xi, 775. London: Cassell & Company, Limited. 1909.—The third edition of this important work has been completely revised by Mr. Jonathan Hutchinson, who has found it necessary to make extensive changes and considerable additions in order to bring the book up to present-day standards. The first volume consists of two parts. Part I, dealing with general principles, contains numerous photographs of aseptic furniture and fittings at the London Hospital and illustrations of sterilisers and surgical instruments. Part II, dealing with abdominal operations, including operations upon the genito-urinary organs and the perineum, contains a number of excellent coloured anatomical plates. The description of the various operations is concise and clear, many obsolete operations being omitted. The accompanying illustrations are excellent. We note with pleasure that each volume has its own index—an improvement which we hoped for when reviewing the second edition of this work.

**Massage in Recent Fractures.** By Sir William H. Bennett, K.C.V.O., F.R.C.S. Fourth Edition. Pp. x, 134. London: Longmans, Green and Co. 1909.—The greater part of the book deals with the treatment of recent fractures, sprains and dislocations by means of massage and movements, the author advocating the treatment to begin in most cases as soon as possible after the injury. He emphasises the value of the hyperæmic, Bier's, treatment as an adjunct to massage in certain forms of stiff joints. The book is well worth reading by anyone interested in the rational treatment of injuries of joints.

**Synoptic Chart of Cardiac Examination.** Arranged by John D. Comrie, M.A., M.B. London: John Bale, Sons and Danielsson.—A very ingenious and informing toy, giving at a glance the principal physical signs of the ordinary cardiac disorders (thirteen in all) on the sliding card system. Thus, when "Aortic Incompetence" is shown in the headline, you may immediately find in the aortic area "diastolic murmur," at the apex "enlarged left heart," in the neck "pulsation of arteries," and at the wrist a pulse tracing typical of the disease. Quite complicated lesions are similarly illustrated. It would be very useful to hang in the student's study or on the walls of the house-physician's sitting-room.

**Pye's Surgical Handicraft.** Fifth Edition. Revised by W. H. Clayton-Greene, B.A., M.B., B.C. (Cantab.), F.R.C.S. (Eng.) Pp. xvi, 592. Bristol: John Wright & Sons Ltd. 1909. —It is nearly ten years since the last edition of this valuable hand-book appeared. The present edition, (fifth), is of necessity a larger book; it is better printed and in better type, and altogether more attractive than the last. Although largely rewritten, the general plan of the book has been mainly adhered to. The chief additions we notice are a chapter on massage, manipulation, and passive movements in the treatment of fractures; one on injuries of the spine, and chapters on the following special subjects: Eye, nose and throat, larynx and ear. There is a large addition to the number of illustrations. The general character of the work is so well known, and has been so often reviewed in the past, that it is unnecessary to refer to it. It is a most useful book to be in the hands of the student when commencing his surgical hospital practice, and should be value to those house-surgeons who have not had much practical experience during their student career. We see the flute-keyed stomach pump still pictured and its use explained. We think it would have been better omitted, and that those for whom the book is intended had better limit themselves to the much safer tube and funnel.

**Differential Diagnosis of Bacteria.** By E. P. MINETT, M.D., D.P.H. Pp. viii, 72. London: Baillière, Tindall & Cox. 1909.—This is quite a useful little hand-book for the student preparing for examination, or for the practitioner who does his own bacteriology. It gives, with simple working directions, the differential diagnosis of the ordinary bacteria, briefly setting out the cultural and staining reactions, and the effects of inoculation. About twenty lines are devoted to each organism. It appears to be reliable and useful.

**Studies on Immunity.** By ROBT. MUIR, M.A., M.D. Pp. xi, 216. London: Oxford Medical Publications. 1909.—It is by no means clear what purpose the publication of this book subserves. It is admittedly a collection of papers published separately in various journals and proceedings between the years 1903 and 1909. It is stated by the author that Paper No. 1 has been "largely rewritten and extended so as to make it more of the nature of an introduction to the subject." Had this excellent resolve been realised there would have been some excuse for the book, but unfortunately Paper No. 1 is by no means to be recommended as an introductory treatise to the subject of Immunity; while Papers Nos. 2 to 11 must be familiar to all workers in this particular branch. As a collection of valuable papers there can be no question as to its erudition, but as a volume on Immunity, where the field is open to the writer who will discourse on his subject with the simplicity of a master, it fails.

**Parcimony in Nutrition.** By Sir JAMES CRICHTON-BROWNE, M.D., F.R.S. Pp. vi, 111. London: Funk & Wagnall Company. 1909.—This essay is written as a criticism on the views, which the author believes to be erroneous, but which have received much attention of late years in consequence of their advocacy by Professor Chittenden. It is maintained that the commonly accepted food standards are false, that the quantities of food stuffs are far larger than is needful, and that we have been partaking of double or treble the amount of proteid food which is necessary. The principles of "Fletcherism" and the "Chewing Song" of the Battle Creek Sanatorium are an outcome of this new doctrine. Sir James has set himself to combat these views: the one set called by Mr. Hubert Higgins "psomophagic," as opposed to the other, the low proteid-feeders, which are masticating and ruminating, or "poltophagic," and he has made a careful investigation of the question: "What is the proper daily proteid intake to meet the needs of the human body?" He concludes as follows: The urgent question for us to-day is not how we may teach the poor to thrive on an attenuated fare, but "where shall we buy bread that these may eat?" We should aim not at parcimony in nutrition, but try to "scatter plenty o'er a smiling land." We should prefer the word "parcimony" to be spelt with the usual "s."

**Parents, Teachers and Schools.** By J. Odery Symes, M.D. Clifton: J. Baker & Son.—These lectures on School Hygiene form a booklet of sixty-one pages, containing the views of one of our hospital physicians on questions which come into his work as physician to the Clifton College, and on which he speaks with some authority. His views are grounded on experience and common sense, and as such are well worthy of careful consideration from the parents and teachers for whom the book was written. His medical colleagues will, for the most part, entirely agree with his suggestions as regards diet and exercise, which will further the physical, mental and moral welfare of boys and girls at school, and tend to counteract the prevailing nurture of fads and the nervous diseases of civilised life.

**Transactions of the American Pediatric Society.** Vol. XX. Pp. viii, 238. New York: E. B. Treat & Co. 1908—1909.—This volume contains no less than seven papers by various writers on cerebro-spinal meningitis and cognate subjects. Flexner and Jobling publish an analysis of 400 cases treated with Flexner's serum (meningococcic), and report that their earlier favourable results have been extended by their larger experience. "The number of complications which arose was small, and the only persistent defect noted was deafness." This occurred in a few instances only. Dunn, with 40 consecutive cases treated by Flexner's serum, compares the results with those obtained by antidiphtheritic serum. Knox and Sladen report on 21 cases with a mortality of 14 per cent. They regard the serum as both antitoxic and bactericidal in action. Holt, in reporting on the cutaneous and ophthalmic test for tubercle, states that as a result of 1,000 observations he regards both as equally reliable, and generally prefers the former. He gives a guarded but, on the whole, favourable verdict as to the value of these tests, and notes that they do not distinguish between a latent and active process. The majority of the speakers who discussed his paper, including Dr. Rotch, expressed similar views as to the value of these tests. The Presidential address by Dr. Kerley on "Public School Education," does not deal with what Englishmen would understand by the term. It is, however, a thoughtful and humourous essay, and in many ways quite applicable as a criticism of elementary education in this country. The following sentence is instructive and somewhat tragic: "A few years ago, when I was experiencing difficulty in getting workmen at $5 a day, a lawyer friend advertised in a daily paper for clerks . . . at a salary of $8 a week. Among those who applied were seventeen lawyers who had been admitted to the bar of New York State." Dr. Kerey's final advice to educationalists is peculiarly applicable to our own country: "That labour is the inevitable lot of the majority, and that the best education is that which makes labour the most productive."

**Golden Rules of Dental Surgery.** By C. W. Glassington, M.R.C.S., L.D.S. (Edin.) Third Edition. Pp. 72. Bristol: John Wright & Sons Ltd.—These rules will be found useful by students, but we think the following points need correction in the next edition. One cannot imagine a qualified dental surgeon using an elevator to remove an upper wisdom tooth (page 26). On page 32: Examination of the pharynx before treating superior protrusion is advised, but as the interior of the nose may be also, or only, at fault, the case ought to be sent to a rhinologist. On page 33: The rule as to extraction of the six-year-old molar requires qualification. On page 35: "To and fro" administration of $N_2O$ should never be used. On page 67: The advice to medical practitioners, *re* the use of gargles and administration of calomel, is out of place in this booklet. On page 68: Much better advise the patient to see a dental surgeon, as the medical practitioner cannot be expected to diagnose dental cases.

**Home Nursing, with Notes on the Preservation of Health.** By Isabel Macdonald, A.R.San.I. London: Macmillan & Co. 1909.—This book of 326 pages is full of most valuable information to those who know it not. Although of Scotch origin, it is written in plain language, and is intended for those women and mothers who cannot always have a trained nurse in the home when sickness visits it. Chapters on home hygiene and on infant rearing add much to its value. The authoress has been accustomed to lecturing in nursing and hygiene, and has dedicated her book to the Lady Superintendent of Nurses at the Royal Infirmary of Edinburgh. We think that the chapter on cooking for invalids might well be studied by many fully trained nurses, and that a future edition would be improved by the addition of an index.

**New and Non-Official Remedies, 1909.** Chicago: American Medical Association. 1909.—This booklet of 167 pages contains descriptions of the articles which have been accepted by the Council on Pharmacy and Chemistry of the American Medical Association prior to January 1st, 1909. Every endeavour is made to prevent admission to the "N.N.R." of any articles which are unscientific in composition as useless and inimical to the best interests of the public or of the medical profession. It is proposed to issue the book annually; a quarterly list of further substances is also to be issued as a supplement to the work. A full index of substances, and of manufacturers, gives additional value to the publication.

**The Open-air Treatment of Pulmonary Tuberculosis.** By F. W. Burton-Fanning, M.D. Second Edition. Pp. xii, 184. London: Cassell and Company, Limited. 1909.—The second edition of Dr. Burton-Fanning's monograph on the open-air treatment of pulmonary tuberculosis contains little that was not in the first edition, but is none the less valuable on that account. The chief additions are found in the chapters on etiology, selection of cases and treatment of convalescents. The first is very good indeed, and contains, shortly and concisely, all the more recent additions to our knowledge, with the exception that no mention is made of the frequency with which tubercle bacilli may be detected in the blood. The views of the "inhalation" school (Cornet and Flügge) and of the "ingestion school," as represented by Calmette and von Behring, are clearly stated, and reference is made to the refutation of Koch's statement by the work of the Royal Commission. The remarks on the frequency with which the fever of tuberculosis is mistaken for influenza are very sound. Other new sections are the notes on Calmette's and Pirquet's tests, where the writer will find many to agree with his conclusion that neither is really to be relied upon. Allusion is made to the use of both X-rays and tuberculin as a means of diagnosis, but the author is of opinion that none of these adventitious aids is to be compared to a careful weighing of the facts of the patient's history, coupled with accurate observation of his condition, if possible extending over a period of several days, as a means of diagnosing phthisis early. The precautions stated to be necessary to obtain correct readings of the temperature when taken in the mouth make it a matter of regret that the author cannot see his way to recommend, unreservedly and only, the taking of temperatures in the rectum, a method which those who employ it find to be entirely free from objection. When we consider the extensive use which has been made of tuberculin as a curative agent, it is strange to find no mention whatever of it; but the writer does not stand alone if he considers that it does not yet fulfil his postulate that "the open-air treatment simply makes use of all the various agents which are *proved* to exert a beneficial influence," etc. Some pages are devoted to a consideration of the work of Sir A. Wright and others on the opsonic index; and the author is evidently a believer in the accuracy of the results obtained, as no mention is made of the many somewhat destructive practical criticisms that have from time to time been made of the methods of estimation, not to mention the deductions to be made from these. The above remarks apply almost solely to the additions in the new edition. The chapters on selection of cases and that on after-treatment are of outstanding merit, and they alone make the book worthy of a place in the library of every practitioner of medicine. Great stress is rightly laid upon the importance of giving up a long period of time to the treatment; this is especially necessary in these days of statistics, when even those who ought to know most about it are inclined to discharge patients as "fit for work" far too soon. There are many extremely practical points in the book, as an instance of which may be quoted the paragraph on the care of the teeth before entering a sanatorium. The book is in every way excellent, and deserves to be widely read.

**Infected Ears.** By F. FAULDER WHITE, F.R.C.S. Pp. 100. London: Yellon & Mansfield. 1908.—The author emphasises the favourable experiences that he has had in the treatment of chronic purulent otitis media by means of the operation generally known as ossiculectomy, together with the means usually adopted for opening and draining the attic, an operation which he terms otectomy, to our minds introducing an unnecessary confusion in nomenclature, which serves no useful purpose.

**E. Merck's Annual Report, 1908.** Vol. XXII. 16 Jewry St., London, and Darmstadt. 1909.—We are again glad to welcome this report of recent advances in pharmaceutical chemistry and therapeutics. It is not a mere trade advertisement, but appears to be an outcome of an honest desire to provide a brief and impartial review of the therapeutic acquisitions of the last twelve months. The spirit of impartiality which it displays deserves all commendation, and to this is greatly due the fact that this Annual is so widely appreciated, and so generally finds a place on the consulting-room table as a work of reference when the latest developments of the pharmacist and the chemist are being sought for. It can be obtained free by any medical man who may apply for it. The subject of organo-therapy occupies over one hundred pages. It is to be regretted that so few of the substances described in the report have as yet proved themselves to possess any reliable utility; many of them are greatly eulogised, but we think that the desire to discover some novelty is often more in evidence than any useful result which can be established, but with such results as have been obtained from the use of thyroid and suprarenal preparations, it would be rash to limit the possibilities of organo-therapy in the future. The second portion of the book consists of some 250 pages on preparations and drugs, many of which are not in common use. The reader will find all the latest views and instructions as to the mode of administration and doses of such drugs as arsacetin, atoxyl, diaspirin, fibrolysin, novaspirin, sajodin, urotropin, and veronal; also an account of the curative sera and bacterio-therapeutic preparations, including the antithyroidin of Mœbius and the pneumococcic serum of Merck. The various indices which follow add much to the utility of the book as a work of reference, and a general index of diseases with indications for treatment may now and then supply a useful suggestion for clinical purposes.

**Clinical Manual of Diseases of the Throat.** By JAMES WALKER DOWNIE, M.B. Second edition. Pp. xviii, 432. Glasgow: James Maclehose & Sons. 1909.—The author has considerably enlarged on the first edition, and, in fact, he has practically re-written his work, though successfully keeping in view the object of producing a practical guide for the senior student and general practitioner, a purpose which has been admirably fulfilled. We are glad to find the author recommends the guillotine for tonsillectomy, but we think fuller and more detailed directions are desirable for enucleation. He omits all reference to compression of the bleeding base in troublesome hemorrhage, and we rather pity the practitioner who, when styptics have failed, has to depend on catching up the bleeding point with forceps and applying a ligature. Intubation is well described, but it is a mistake to advise the thread loop being cut if this can be avoided, since with the shorter period of retention of the tube, with the use of antitoxin, the thread can generally be retained for extracting the tube much more readily and safely than by the extractor. We are sorry to find no indications to guide the practitioner in his choice between a tracheotomy and intubation. But the manual is, speaking generally, complete, and very sound in its teaching; the coloured plates and black and white illustrations are very good and useful, and the printing excellent.

**Transactions of the Royal Academy of Medicine in Ireland.** Vol. XXVII. Dublin: John Falconer. 1909.—The solemn dulness usual in volumes of Transactions is in this case relieved by an entertaining article on "Baths and Mineral Waters, and Some Fallacies connected therewith," by Dr. Walter G. Smith. He maintains that the answer to the therapeutic problem is not to be found in trivial details of the specific virtues of the different springs, but that "a Carlsbad cure is a very complex therapeutic agent. It is a rest cure, a diet cure, an air cure, a music cure, an exercise cure, a mind cure, and a body cure."

